# An Anomaly in Obstetrics

**Published:** 1879-01

**Authors:** A. H. Wilson

**Affiliations:** Jonesboro, Texas


### AN ANOMALY IN OBSTETRICS.

By A. H. WILSON, M.D., Jonesboro, Texas.

A very remarkable case of obstetrics, occurred in my practice, and at the request of some friends in this section, I prepared and published a statement of it more particularly for the people in this immediate vicinity, the circulation of which has resulted in quite an extensive correspondence from a distance. Among the number of correspodents are several of our most eminent practitioners and professors; several of whom have requested me to prepare a statement to be published in a medical journal, that the members of the profession generally may have an opportunity of investigating it. The unusual phenomena presented during the progress of the case, with the singular abnormal condition of the uterine contents, constitutes it a very remarkable case indeed; one of unusual initerest to the medical student, practitioner and professor.

Case: Mrs. M. was near her sixth confinement, on the 12th of April, 1878; she did the washing for a large family; she was twenty-eight years old, very fleshy, and below the medium height.

At 6 A.M., of the 13th, she was attacked with pains; they continued through the day and night, and up to the time I was sent for, which was 6 P.M., the 14th. The pains were sharp, short and very ineffectual. Her strength failed gradually from the commencement of the pains, and at the time of my arrival, the pains were not so frequent, but seemed to be more forcing; the patient was very feeble, with an anxious expression of countenance, restless, and turning from side to side, sitting up, very talkative, the surface cool and damp, pulse feeble, quick and hard, and breathing quick and laborious. At 7 P.M., I made an examination, per vagina, finding the os uteri dilating, and a natural presentation. I assured her that labor was progressing as faborably as could be desired, urging her to remain quiet; the pains were now more effectual, and she was more restless as the labor advanced. At 9 P.M. the os was open as large as a half dollar; the membrane could be distinctly felt at this time, and the progress of labor was so favorable, that I used every means to induce the patient to remain quiet; reminding her of the importance of controlling herself, and the danger of the course she was pursuing. She replied:

"I know all you say is true, but I cannot help it; I feel like I was smothering all the time."

She continued to toss from one side of the bed to the other; during which time the progress of labor was slow, but regularly advancing. At 11 P.M., the liquor amnii passed; the head at this time passed into the vaginal canal; the pains from this time were very effectual and regular; the patient was very restless; indeed from this time it seemed she could not control herself, and neither could she be controlled by any means we could employ. She remarked:

"I cannot be still; I try, but cannot help moving;"

And said she must get out of bed, that she was smothering, and if she did not get up she would die. My protest availed nothing; she was taken up and placed in a chair; in a few moments she complained of being sick, and wished to be helped back to bed; which was done; she was in bed but a moment, when she fainted, but soon recovered. Her skin was cold and clamy; pulse very weak and thread-like; the expression of her countenance was such that I can never forget it; so wild, and yet imploring. Before I could determine my course, the child, weighing eleven and a half pounds, was expelled. Another surprise was then experienced, for not a drop of blood passed with the child. Before the cord could be tied the patient fainted; again I directed the usual restoratives to be used; she soon revived; so soon as I tied, and severed the cord, and handed the child to the nurse. I examined the pulse, and found it scarcely perceptable; could not count it; surface cold, and covered with a clamy sweat. I supposed there existed concealed hemorrhage; I was sure that if hemorrhage was the cause of her condition, it was concealed; I sought for it in the abdominal cavity; there was no evidence of it there; I felt for the womb, but could not find it, though the abdominal wall was very flaccid; not finding the womb, I grasped the cord with the left hand, and passed the right into the vagina, using the cord for a conductor. I pushed the hand into the uterus, finding the placenta attached a little beyond the cervix; I detached it with the finger; the patient was so uncontrolable that I withdrew my hand, making gentle traction on the cord; there was no movement of the placenta; I feared I had not detached it entirely; I introduced the hand as before, passing it into the uterine cavity, grasping the afterbirth, I found it free, and lying in a placid uterus; indeed the walls felt like a wet buckskin sac; I turned the hand around, and felt a slight contraction—withdrawing the hand, the placenta followed. At this time there was a slight pain, which expelled the afterbirth with some force, as it dropped clear of the vulva, and fell on the bed.

Strange as it may seem, not a drop of blood escaped from the vagina up to this time; the bed was not stained, nor was there any in the uterine cavity when I introduced my hand; the absence of blood, up to this time, I assure you perplexed me greatly. Connected to the placenta, by adhesion, as it seemed, was a membranous sac; the placenta appeared normal, rather pale in appearance; the connection was very distinct and easily traced by a faint white line. The appearance of the sac and placenta, as they were lying on the bed, was such as we sometimes see with twins. With the first, the membrane burst, and the child is pushed out without covering; the second passes with the afterbirth attached, the membrane containing the child and liquor amnii remaining unbroken. After examining the sac, and finding it contained nothing but fluid, I ruptured it, and emptied the contents—(blood,) four or five quarts. The sac was of a membranous substance, very similar to the fœtal amnion. Taking up the placenta, I felt something in it which I at first could not account for. I examined for the point that this substance passed into its present location; finding none, I noticed that there was a delicate filament extending from the vagina to within the placenta; in order to learn what it was, as it was concealed in the placenta, I with the scalpel sliced off the placenta, and found the tumor imbeded therein just as a peach seed is found in the peach. The tumor was very smooth, and nearly the size and shape of a goose egg, with this filament, attached to it. In pressing it, the resitance was similar to that of a rubber ball. I returned this tumor back into the womb with facility, using only one hand for the purpose. The patient was sinking all this time; (from the birth of the child to the expulsion of the afterbirth was perhaps five or ten minutes;) I had no stimulants to give her, therfore sent for Dr. Pope, who arrived 4:30 A.M., on the 15th. He made an examination, per vagina, and with the finger reached the os, which he found nearly in its natural position. At this time there was a slight discharge of what he called "bloody water." With this exception, there was no discharge from the uterus from the birth to her death; which occurred at 5 A.M. on the 15th. Forty-two hours elapsed from the first pains to the expulsion of the placenta.

I have given all the important particulars of this very curious case, and give it to my professional brethren with the hope that it may not be uninteresting; and to the inquiring or investigating mind, that the curious phenomena presented during the progress of the case, and the singular abnormal condtions of the secundines, will be clearly and practically solved.

The points of interest involved in this case are:

First. Of what, and how was the blood sac formed?

Second. What was the cause of hemorrhage?

Third. When did the hemorrhage begin?

Fourth. How did the blood pass into the sac?

Fifth. What was the substance found in the placenta?

The solution of these questions will account for the abnornal condition found exhising in the flaccid uterus and its contents. In order to arrive at a rational explanation of these questions, we should take them up in the order they bear on the case.

First. Of what was the sac formed?

Answer. The placenta being formed of the chorion, the ovum, at a certain time, burst its chorion covering; the chorion shrinks and falls back to the placenta, as some physiologist express it, forming a kind of cushion for the placenta. Now it is not possible for the decidua, after serving its time as the covering for the ovum, falling on the placenta, and forming around that organ as it does, to be the material from which the blood sac was formed.

Second. What was the cause of hemorrhage?

Answer. It was external injury received on the 12th.

Eighteen or twenty-four hours before the pains commenced, a woman bending over a wash tub, perhaps a wash board in it, so near her confinement, would most likely detach a portion of the placenta from its uterine connection. Now in this case, I suppose it was only detached in the center of the placenta; the rim, or edge, remainging undisturbed; this being the case, we can understand why there was no discharge of blood at the birth of the child, and none found in the uterus, for the blood had all passed to the sac, and was sealed up therein.

Third. Where did the hemorrhage begin?

Answer. I believe it commenced to flow from the uterine sinuses at the time the placenta was partially detached. The detachment was but a small portion, hence the system was not affected by the loss of blood, until the placental attachment was further broken up, by the accumulating blood between it and the uterine wall, which by 6 A.M., the 13th, was sufficient to cause the system to feel its loss as was evident by a growing weakness from this time to the end of the patient's existence.

Fourth. How did the blood pass into the sac?

Answer. The placenta, being a vascular, fleshy excrescence, attached to the uterine wall by adhesion, is easily detached. Now, in this case, it is but reasonable to suppose that a portion was so detached by an injury received on the 12th, leaving a portion of the uterine sinuses open, through which blood flowed into the detached portion, the weight or force of blood being sufficient to detach the placental adhesion, according to its requirements. The placenta being of a spongy and distensible substance, gave way and permitted a large amount of blood to be there concealed. By the Endosmotic process the blood was conveyed through the placenta, and meeting with a distensible membrane (which I suppose was the reflex decidua) the blood, pressing upon this membrane, caused it to distend into a sac, adjusting its capacity to the demand made upon it by the blood, which had now made a channel through the spongy substance of the placenta. Without any obstruction the blood flowed from the veins, which permeate the womb, they having been ruptured by external violence, or from the blood rushing through them with such force as to rupture their now exposed endangium. Yet, the flow from the rupture was somewhat obstructed until the process of endosmosis was established and the channel through the placenta opened to the distensible sac, then the blood flowed unobstructed through the open mouths of the uterine sinuses.

That the supply was exhausted is evident from the condition of the patient, and the absence of any flooding during the process of parturition. That this was a gradual drain, for a considerable time, is made to appear from the restless and gradually sinking condition of the patient from 6 A.M. of the 13th, which kept regular pace with the flowing blood as it seemed.

Fifth. What was the substance formed in the placenta?

Answer. From the firm, elastic feel of the tumor, we might suppose it to be a polypus, or a fibroid, but from the fact that it was connected to the uterus by a slender filament, it could not be either of these. I am inclined to the belief that it was a mole, the result of a blasted ovum. When it perished it lodged in the membrane of the womb. Instead of taking the usual course, its place of lodgment happened to be the point where the placenta was to settle on the uterine wall. The tumor not being dependent on the placenta for its support, but receiving its support as the placenta from the womb direct by a delicate filament. The placenta, in its growth and adhesion to the womb, enveloped the tumor, and lifting it from its lodgement, thereby encasing its own substance.

This is the only solution I can give of the tumor, as it was completely encased in the placenta. I have now given my statement and views of this very peculiar case, and hope my professional brethren will favor me with theirs, either through the JOURNAL or direct to me.

